# Harm reduction, methadone maintenance treatment and the root causes of health and social inequities: An intersectional lens in the Canadian context

**DOI:** 10.1186/1477-7517-8-17

**Published:** 2011-06-30

**Authors:** Victoria Smye, Annette J Browne, Colleen Varcoe, Viviane Josewski

**Affiliations:** 1 University of British Columbia, School of Nursing. T201-2211 Wesbrook Mall, Vancouver, B.C. V6T 2B5, Canada

## Abstract

**Background:**

Using our research findings, we explore Harm Reduction and Methadone Maintenance Treatment (MMT) using an intersectional lens to provide a more complex understanding of Harm Reduction and MMT, particularly how Harm Reduction and MMT are experienced differently by people dependent on how they are positioned. Using the lens of intersectionality, we refine the notion of Harm Reduction by specifying the conditions in which both harm and benefit arise and how experiences of harm are continuous with wider experiences of domination and oppression;

**Methods:**

A qualitative design that uses ethnographic methods of in-depth individual and focus group interviews and naturalistic observation was conducted in a large city in Canada. Participants included Aboriginal clients accessing mainstream mental health and addictions care and primary health care settings and healthcare providers;

**Results:**

All client-participants had profound histories of abuse and violence, most often connected to the legacy of colonialism (e.g., residential schooling) and ongoing colonial practices (e.g., stigma & everyday racism). Participants lived with co-occurring illness (e.g., HIV/AIDS, Hepatitis C, PTSD, depression, diabetes and substance use) and most lived in poverty. Many participants expressed mistrust with the healthcare system due to everyday experiences both within and outside the system that further marginalize them. In this paper, we focus on three intersecting issues that impact access to MMT: stigma and prejudice, social and structural constraints influencing enactment of peoples' agency, and homelessness;

**Conclusions:**

Harm reduction must move beyond a narrow concern with the harms directly related to drugs and drug use practices to address the harms associated with the determinants of drug use and drug and health policy. An intersectional lens elucidates the need for harm reduction approaches that reflect an understanding of and commitment to addressing the historical, socio-cultural and political forces that shape responses to mental illness/health, addictions, including harm reduction and methadone maintenance treatment.

## 

There is considerable evidence that harm reduction approaches are effective in reducing the harms associated with drug use [1-3]. As Pauly notes, "harm reduction as a philosophy shifts the moral context in health care away from the primary goal of *fixing individuals *towards one of *reducing harm*" (italics ours) (p.6) [4]. However, although harm reduction opens opportunities for promoting the health of people who often are stigmatized through social responses to problematic substance use, harm reduction interventions do not necessarily address the root causes of substance use and attendant social conditions that influence inequities in health and access to health care for this population - "inequities [that] are exacerbated by lack of quality housing, poverty, unemployment, lack of social support and education" (p.8) [4]. Harm reduction approaches that fail to address the multiple intersections that influence peoples' health and well-being and their experiences of and responses to mental health and addictions care may also fail to improve health in a meaningful way [5].

In keeping with the perspectives of Hankivsky, Cormier and de Merich, we believe that peoples' health and experiences are shaped by a number of intersecting variables associated with social identity, such as "race/ethnicity, Indigeneity, gender, class, sexuality, geography, age, disability/ability, immigration status, religion etc. - variables that also have been associated with oppression (e.g., racism and classism) and consequent disadvantage (e.g., poverty and homelessness)" (p.7-8) [6]. For example, in the study in which this paper is grounded, the client participants were Aboriginal, and 'race' was relevant to all experience - the race-based privilege of oppression was present in the everyday reality of peoples' lives, including the experiences of accessing and delivering MMT. Yet 'race' could not be neatly shifted apart from processes of racialization, issues of gender, class relations, and other social relations that structured peoples' lives such as their education level, employment status, health, and well-being. As Bannerji notes, "[r]acism is after all a concrete social formation. It cannot be independent of other social relations of power and ruling which organize the society, such as those of gender and class..." (p.128) [7] - the relationship between these variables is complex and interdependent [6,8-10], occurring within and intersecting with societal contexts. Anderson and Reimer Kirkham note that to understand the meaning of health within a sociopolitical and cultural context, there is a need for an elucidation of "the intersectionality and simultaneity of race, gender, and class relations, the practice of racialization, the connectedness to historical context, and how the curtailment of life opportunities created by structural inequities influences health" (p.63) [11].

Intersectionality is increasingly being used in health research as a lens for highlighting the inter-related and co-constructed nature of social locations and experiences[6,12,13], and for understanding differences in health needs and outcomes in mental health and addictions and harm reduction [14]. As Weber and Parra-Medina note, inequities are often obscured when models of practice focus on individual bodies [and behaviour] rather than taking into account "the social structural context as the locus of a population's health" (p.187) [12]. Grounded in critical feminist theoretical perspectives, intersectional analyses are useful in drawing attention to the dynamics of the intersections between problematic substance use, other aspects of social identity and different forms of oppression associated with social and structural contexts that can guide us in the pursuit of addressing the multiple inequities and intersecting multiple stigmas associated with drug use.

In this paper, we focus on harm reduction and methadone maintenance treatment (MMT) to illustrate how social change can be promoted using an intersectional lens to examine harm reduction and MMT and mental health and addictions more broadly. We use findings from a partnership-based research project conducted in British Columbia, Canada, entitled, *Aboriginal peoples' experiences of mental health and addictions care: Toward improved access*, to elucidate how an intersectional lens can provide a more complex understanding of harm reduction and MMT - how harm reduction and MMT are experienced differently by people dependent on how they are differently located (e.g., living in poverty and homeless and/or near homeless). Using the lens of intersectionality, we refine the notion of harm reduction by specifying the conditions in which both harm and benefit arise and how experiences of harm are continuous with wider experiences of domination and oppression. This paper is not meant to be an indictment of harm reduction or MMT; rather, we use an intersectional lens to elucidate the need for harm reduction approaches that reflect an understanding of and commitment to addressing the historical, socio-cultural and political forces that shape responses to mental health and addictions and harm reduction.

## Background

### The Complexity of Problematic Substance Use, Addiction and Associated Stigmas

In this paper, our focus is on issues pertaining to problematic substance. In particular, our research has focused on people who identify as Aboriginal and who are most impacted by the marginalizing conditions of persistent social and structural inequities - poverty, homelessness, unemployment and so on. From the outset, we want to be clear that problematic substance use is not always associated with mental illness, homelessness, Aboriginal identity etc., however, the issues discussed in this paper represent insights provided by conducting research with Aboriginal people whose lives have been most influenced by these sociopolitical circumstances.

In keeping with the perspective of Reist, Marlatt, Goldner, Parks and Fox, we understand the phrase 'problematic substance use' to encompass the concepts of potentially harmful substance use behaviours or patterns (e.g., impaired driving or the use of substances during pregnancy) that are not clinical disorders and 'substance use disorders'(i.e., clinical disorders defined by the DSM-IV, including dependence or addiction) (p. 4) [15]- with a spectrum of use from 'beneficial' to 'non-problematic' to 'problematic use' (p. 8). From this perspective, substance use is not problematic for everyone, and one substance may present a problem for the individual where another may not. In addition, substance use can be stable at one point in time and move gradually or rapidly to a different point (p. 8) [15].

The associated harmful consequences of problematic substance use may include physical illness, including increased risk of infection (e.g., HIV, Hepatitis C and other blood borne infections due to sharing drug paraphernalia); family breakdown; economic issues; criminal involvement; and a high risk of overdose leading to death, and death by violence [16-19]. In addition, the issue of stigma is a highly pertinent concept intersecting with [or contributing to] the harms associated with problematic substance use (p.5) [20,21].

In this paper we take up Goffman's (1963) notion of stigma as an attribute associated with 'difference' that is deemed to be a less desirable difference by one person (the stigmatizer) in relation to another person (the stigmatized) - a difference, which at its extreme, might deem the person as bad, dangerous or weak (stereotyping) (p. 12) [22]. Further as Link and Phelan argue [23,24], stigma is created through five interrelated and converging social processes, for example, in the case of drug use: i) labeling of the person with problematic substance use as different, e.g., the 'drug addict' or 'junkie'; ii) negative stereotyping by linking 'difference' with undesirable characteristics and fears such as drug users as "dangerous"; iii) 'othering' by creating "them" (the labeled person) and "us" categories; iv) status loss, blame and discrimination of the labeled person; and, v) creation of power dynamics in which power is experienced by the labeled person's ability to access to key resources, such as money and social networks/institutions [25,26]. Thus, problematic substance use as a category of 'difference' often leads to stigmatization based on the beliefs that underpin its perceived origins and an experience of and the ability of the labeled person to resist stigma (or not) dependent on their social location and perceived power.

Although public attitudes vary towards people with problematic substance use and many people acknowledge that people with drug use issues often come from difficult circumstances, i.e., that there are social and structural issues influencing use, there remains a strongly held view that "drug addicts" are to blame for their drug use [27]. For example, Henderson et al. (2008) concluded that while staff in their hospital study were committed to providing care to people with problematic substance use, their training and experience led them to treat them differently from other patients - particularly notable in the area of pain management (as cited in Lloyd, (2010)) [27] where physicians, as one example, are trained to the on alert to "drug seeking" behaviour in this population. As Lloyd notes, in our society, the identity as "addict, tends to take center stage to the obscuration of all other facets of identity and personality..." (p.13)[27].

Additionally, individuals who are "addicted" or dependent on substances often lead "chaotic and stressful" lives and may have additional co-occurring and stigmatizing mental health and other health issues; these intersect with social issues associated with their substance use that make diminishing or abstaining from substance use extremely difficult (p.16) [17,21,26,28,29]. Chaos and stress are most often related to intersecting factors, such as poverty, unemployment, housing issues and stigma and discrimination [17]. Lack of housing and/or meaningful employment have also been shown to *contribute to *substance use and addictions. In this paper, we use an intersectional lens to shift attention from the individual to the social and structural inequities that may influence substance use, and health and well-being for those with *problematic *substance use. For example, substance use needs to be understood as sometimes overlapping with violence and mental health issues, and those problems need to be seen within the context of social and structural determinants of health to ensure the provision of integrated care [30,31].

### Examining Harm Reduction and MMT through an Intersectional Lens

Given the complexity of problematic substance and associated stigma as presented above, the complexity of issues that shape practices and policies related to MMT are best understood under the pragmatic philosophy of harm reduction (p.18) [17]; an approach that represents a continuum of services that embody a philosophical, pragmatic and compassionate approach to providing care while minimizing the negative harms associated with substance use, understanding that not all people have the same ability to change, the same level of drug use, or even experience the same harms [5]. Two central underlying values of a pragmatic perspective to harm reduction is i) that all life activities carry risk *and *ii) that elimination of drug use is not necessarily attainable or desirable [4]. This approach to harm reduction is goal-oriented, humanistic [32] and in keeping with a cost benefit awareness [5,33]. Humanistic values explicitly highlight the values of respect, worth and dignity of all persons, therefore, there is a focus of "nonjudgmental acceptance of persons [who use illicit drugs] as worthy of respect without judgment of drug use" (p.6) [5]. The active participation of the client is acknowledged as important in harm reduction programs [5,32].

Central to a harm reduction approach is "a focus on reducing the negative consequences of substance use for individuals, communities and societies...rather than focusing on decreasing or eliminating substance use" (p.6) [5]. Harm reduction occurs gradually in a step-by-step progression toward decreased levels of overall harm [33]. In keeping with this perspective, harm reduction is one aspect of a comprehensive approach to the harmful consequences of drug use, recognizing that there are many different strategies and programs of harm reduction that meet diverse clients' needs. Health care professionals using a harm reduction approach meet clients "where they are at" in terms of their ability to change (p.14) [33], and work collaboratively with clients to establish goals and develop a client-centered plan of care [17]. Lastly, a harm reduction approach is underpinned by a commitment to change policy and/or to be integrated into existing health policies. Examples of specific harm reduction strategies include needle exchange programs, safe injection sites, distribution of condoms and dental dams (all products should be freely available and offered without cost), bleach kit programs for cleaning syringes, distribution of clean crack kits, safer sex education, safer drug use and education, outreach programs for high-risk populations, law-enforcement co-operation, prescription of heroin and other drugs, and methadone maintenance treatment, among others. However, most of these efforts deal directly with the harms that emanate from individual drug using and sexual practices, and deal less with the harms associated with the root causes of problematic substance use (violence, poverty, racism, historical trauma and so on), and the harms associated with drug policy (such as criminalization, incarceration, poverty). Although all important strategies, the root causes of illicit drug use are not addressed. MMT as a harm reduction strategy is an exemplar of how an intersectional lens can elucidate the multiple intersecting factors that shape experience.

As a substitution/maintenance therapy, MMT is considered the "gold standard" (p.6) [34]. "Systematic reviews have identified MMT as the most effective form of treatment for opioid dependence in terms of treatment retention and decreases in the use of illicit opioids" [[35-37] as cited in 21]. Methadone is a long-acting synthetic opioid that binds to the opioid receptors in the body. Being an opioid agonist, it can significantly reduce the rates of withdrawal and cravings associated with opioid dependence [34]. Due to the fact that it is a long-acting drug, there is no euphoric effect, a fact that contributes to lower rates of relapse [16,17,34]. However, as Caplehorn et al. note, one of the greatest benefits of MMT is its well documented decrease in mortality for individuals in treatment as compared to those who use opioids who are untreated [[38] as cited in 21].

According to recent guidelines developed by the RNAO, that are based on a systematic review of the literature, and according to Reist, MMT should ideally encompass an interdisciplinary effort with three components: methadone prescribing, methadone dispensing and a range of comprehensive psychosocial services and supports such as counseling services and supports related to housing, employment, education, mental health, or life skills and access to other health services such as perinatal care and health promotion activities [17,21]- care that takes into consideration the biopsychosocial context of the individual client.

Yet, MMT is often applied within biopsychosocial models in ways that encompass varied strategies but ignore the intersecting social and structural issues that give rise to opioid addiction, resulting in particularly serious consequences for some groups of people - approaches that do not focus on the social forces and contexts that shape people's health and lives, including "the situatedness of social inequality in history and place, and its operation at the macro social structural as well as micro individual level" (p.187) [12]. MMT often involves regulating or managing the social order and 'marginalized' subjects, but fails to deal with the root causes of injustice that give rise to drug use. For example, harm reduction approaches, including MMT, that do not reflect the simultaneous interactions between substance use, gender, class, violence and trauma as complex and interdependent, fail to address the unique needs of women [30,31]. "Substance use and mental health problems frequently co-occur among women who are survivors of violence, trauma, and abuse, often in complex, indirect and mutually reinforcing ways..." (p.32) [31]. In addition, HIV infection due to injection drug use is far more prevalent in women, accounting for 19.2% of all AIDS diagnoses in adult women compared to 3.9% in men [39]. Harm reduction services need to attend to specific needs of women and integrate an intersectional analysis into drug policy and harm reduction frameworks [30]. There is a need to apply what we know about differing patterns, health impacts, pathways to problematic substance use and related experiences in the design of harm reduction service provision and policy, including MMT. An intersectional lens draws attention to how and why MMT needs to reflect approaches that address the multiple inequities, such as those associated with living with mental health and addictions issues, a history of trauma and violence, homelessness, and poverty - to name a few.

In addition to the above issues, the historical and structural inequities that have shaped the health and well-being of Aboriginal people in Canada have resulted in greater risks of experiencing violence, trauma [40-42] and substance use [43]. Yet little is known about the experiences of Aboriginal persons who access mental health and addictions services (mainstream and Aboriginal). In 2006-2009, we conducted a study in partnership with a team of Aboriginal and non-Aboriginal researchers, community agencies and leaders in mental health and addictions and community members to explore Aboriginal peoples' experiences of mental health and addictions care in an urban Canadian context to inform the design of safe and effective [mental] health and addiction services. In Canada, the term 'Aboriginal' is often used to refer to diverse groups of indigenous people who include First Nations, Métis, and Inuit people.

## Methods

### Study Design and Data Collection

A qualitative design using ethnographic methods of in-depth individual and focus group interviews and naturalistic observation was used. Study participants were Aboriginal clients from diverse Nations (as they described themselves) including, Nisga'a, Plains Cree, Cree, Kwagiulth, Cowichan, Blackfoot, Métis, Gitxsan, Dené, Saulteaux Cree, Ojibway, Sioux, Coast Salish, Haida, Sto'lo, Sarcee and Six Nations (n = 39; individual in-depth interviews (n = 18: 8 males, 10 females) and three focus groups (n = 21: 11 males, 10 females) who accessed mainstream and other mental health and addictions services and health care providers, Aboriginal and non-Aboriginal (n = 24; individual in-depth interviews) working within those settings. Ethical approval was sought and obtained by both the Behavioural Research Ethics Board of the University (BREB #H06-80439) and the local ethics committee of the regional health authority. In addition, the study was guided by ethical guidelines of the Royal Commission on Aboriginal Peoples (1993), and the principles of Ownership, Control, Access, and Possession (OCAP) for research with First Nations [44,45].

Purposive and theoretical sampling was used to recruit Aboriginal clients and health care professionals from mental health and addictions settings. Because the purpose of the study was to inform an understanding of how to improve mainstream mental health and addictions services so they are more responsive to the needs of Aboriginal clients, the settings chosen were five community-based mental health and primary health care agencies. Eligible client participants were persons who had no cognitive impairment and identified as 19 years or older, and Aboriginal persons accessing mental health and/or addictions services within these settings. Health professionals who were interviewed were working within the research sites and included the designations of mental health nurse (RPNs, RNs, LPNs), community outreach worker, psychologist, psychiatrist, social worker and support worker. Recruitment was facilitated through 'liaison' people on site as well as through informational study pamphlets that were approved by ethics and posted at the study-sites. The qualitative interview/focus group guides for client participants prompted exploration in the following areas: the reasons for seeking care in this particular setting; assumptions and expectations about the care; experiences of seeking care; and, interest in Aboriginal traditional healing practices. The guide for health care providers prompted exploration related to their experiences providing care to Aboriginal clients and their understanding of why clients seek care in their setting. Interviews occurred within the mental health and/or addictions care setting or within an informal setting and ranged between 30 and 60 minutes. With permission, interviews and focus groups were audio-taped and transcribed. An honorarium of $30 was provided as a way thanking participants for their time. All participants were assured complete confidentiality and provided written informed consent to the study.

### Data Analysis

Using an interpretative thematic analysis, data was analyzed in a multi-step process using comparative coding strategies [46,47]. Using NVivo, a computer software program, transcripts were first coded in 'chunks' of data as a means to organize and group the data. As new data continued to be gathered, whole interviews were read repeatedly to identify recurring, converging and contradictory patterns of interaction, key concepts, preliminary themes, illustrative examples and linkages to theory [47]. In addition, coded transcripts were compared to identify similarities and differences in the coding process. In this way, initial coding strategies were revised and refined as part of regular reflective discussions with the research team. Finally, exemplars from coded categories and themes were retrieved using NVivo and compared within and across transcripts. At this point, interpretations were reviewed using a sub-sample of participants to check descriptive and interpretive validity. Resonating with participants' experiences of their complexity of life, the findings of this study were discussed using an intersectional lens - as a set of complex interrelations rather than a set of discrete variables. For example, one of the core findings which we discuss in this paper underscores the importance of understanding how harm and benefit are differentially experienced by clients of mental health and addictions services dependent on their histories and social location/position.

## Results/Discussion

In this study, client participants presented with significant levels of co-occurring illnesses including schizoaffective disorder, mood disorders, depression, anxiety, suicidal ideation, alcohol and drug use, HIV, Hepatitis C and PTSD associated with complex trauma. Several participants were residential school survivors and most had long histories of trauma, beginning in early childhood and for many, continuing into the present. These factors have been long understood to be associated with mental health and addiction issues. For example, residential schools which included industrial schools, boarding schools, student residences, and hostels, located throughout Canada, the last of which closed in 1996, have been the most often cited cause of the mental health concerns of Aboriginal people in Canada. Although residential schooling was not uniformly negative for all people,^5 ^its overall impact has been devastating [48-53]. In response to this understanding, in 2006, the federal government announced the approval of the Indian Residential Schools Settlement Agreement and the new Truth and Reconciliation Commission [54].

Many of the client participants in this study reported being on methadone, an aspect of the study, we report on in this paper. Further, all of the health care providers worked with clients who had previously accessed MMT or were attempting to access MMT. Using an intersectional analysis, we use the findings of this study to underscore the importance of understanding how harm and benefit are differentially experienced by clients of mental health and addictions services dependent on their histories and social location/position.

The key findings were that a) stigma and discrimination intersected with other disadvantages to profoundly shape people's lives and their access to and experiences with MMT, b) the policy context of MMT constrained people's lives, with significant consequences and these experiences and consequences varied with people's social locations, and c) in concert with poverty and other disadvantages, these constraints contributed to housing instability and homelessness for many. Although harm reduction is based on the values of non-judgment and non-coercive approaches to service delivery [5] and there are many positive outcomes associated with MMT, many of the participants in this study experienced 'harm' associated with "the intersectionality of disadvantages" (p.763) [55].

### Stigma and Discrimination

In keeping with the findings of several authors [21,25,56], the attitude of providers was cited as a barrier to access to care in particular settings by several client and health care professional participants. Our findings provide a glimpse into how stigma and discrimination shape access to MMT. The following interview exemplar illustrates the stigma experience of several client participants (CP),

And its easy to kick a wounded dog, I mean, you know, I mean that's what happens down here, [service providers] don't mean to do it, they don't get up in the morning with a plan to go 'I'm going to go kick ten junkies today,' they don't do it, its just as the day builds, as the day builds they just desensitize, year after year they get desensitized to needs and then they just start dealing with what the immediate needs are.

For this participant, his identity as a "junkie" intersected with a perception of provider (physician) desensitization and/or stigmatization of the "junkie" to explain discriminatory treatment within the site where he accesses methadone. Although this may not have been a case of enacted stigma, i.e., where a person is actively discriminated against [22], this participant may have perceived stigma [22,57] because of the negative thoughts and feelings associated with an expectation of stigma and discrimination e.g., through fear, shame and guilt. It is not uncommon, for example, for clients to experience "MMT as punitive and shaming rather than therapeutic even when the professional may be trying to follow guidelines designed to protect the client" (p. 15) [21]. Regardless of the dynamic or form of stigma, stigmatization is a powerful force that often interferes with access to MMT [21,27,56]. Indeed, research has shown that 'drug user' status can be a barrier to accessing health care and can affect the quality of care received [4,21,56,58,59]. A slightly different experience of discrimination is expressed by another client in the following,

Within the system there is some prejudice people in there and I try not to get too mad with them when I find out that they're prejudice, they don't like Natives and they don't like drug addicts.

For several participants in this study, in addition to substance use as an axis of discrimination, stigma (enacted or perceived) also was attached to an expectation of racialization, a process that is neither neutral nor without consequence. Given their multiple social locations, many people in this study expressed uncertainty about why they were treated poorly by some providers. For example, living as an Aboriginal person in Canada carries with it the "burden of history" [60], and prejudice and racism continue to manifest as new forms of colonial processes and practices erupt; however, persons living with mental illness and/or substance use issues and/or HIV/AIDS and/or Hepatitis C also live with stigma and prejudice associated with those diagnoses [26,61-63] and consequent life circumstances, such as poverty and incarceration. Sadly, the social construction of identity/identities (including disease or illness associated and group identity (p. 3) [26]) interferes with both the ability of people to access and remain in MMT. In keeping with the perspective of Stuber, Meyer, & Link [64], in our research, we have found that analysis of the issues using a singular focus on racism or classism or problematic drug use (as examples of oppression), misses how the meaning and experiences of stigma and prejudice intersect with other important variables to create new forms of discrimination. The stigma associated with drug use is usually only one aspect of an intersecting set of stigmas (p. 47) [27].

Applying an intersectional approach to analyses of experiences of stigma and discrimination has numerous advantages. It acknowledges the complexity of how people experience stigma and discrimination and recognizes that the experience of discrimination may be unique. It also takes into account the social context of the group. It places the focus on society's response to the individual as a result of the confluence of various factors and does not require the person to slot themselves into rigid compartments or categories, i.e., it captures more fully the reality of stigma and discrimination as it is experienced by individuals. This approach allows the particular experience of stigma and discrimination, based on the intersection of factors involved, to be acknowledged and remedied. Attention to multiple disadvantaged social statuses is important to identifying the root causes of health disparities [65] and to designing effective interventions [64].

In the following interview example, a provider (P) working in a harm reduction setting discusses methadone maintenance treatment,

Those on the methadone program...their ultimate objective is to get on methadone and stay on methadone and stay off heroine and then they can use other drugs and there's no consequence to that, other than its affecting their health and it affects the, you know, the methadone and so on ... and because I'm an addictions counselor *I have a hundred and twenty patients on the methadone maintenance program*. So those patients are referred to a counselor for support and for counseling and also to deal with any other substance abuse that they may be experiencing. In about eighty-five percent of the cases those on the methadone program have a dependency on crack, cocaine or some other drug so my role is to do an assessment and refer them to day programs or treatment centers or to out patient counseling to help them more in a harm reduction philosophy... My preference is abstinence, abstinence because of the health, you know, it promotes health...

This excerpt reflects the policy context in which MMT is situated, i) a shoestring approach is supported (120 clients), ii) there is an absence of attention to the social determinants of health, and iii) policies are constrained by the criminalization of drug use. It obviously also reflects the attendant discourses taken up by some health care professionals working in the field. Although our observations of the care provided in this setting suggest that the community of professionals within the organization, including this individual, generally were committed to the provision of compassionate non-judgmental care within a harm reduction framework, the ideology projected by this provider belies a frustration with MMT and drug use more broadly - a reflection of the perspectives of many people in broader society.

Today, many people believe that MMT perpetuates drug use because of the misconception that it merely replaces one addictive opioid with another rather than seeing it as a treatment for opioid use [32]. As Cheung observes, this school of thought often is associated with the idea that abstinence-oriented treatment is the only way to achieve a "drug-free" state in society [32]. This ideology is also perpetuated in treatment programs that do not accept clients on methadone. As one client participant noted, "Yeah, I think that they should put more treatment centers out there that are accessible to methadone [patients]...because a lot of them don't accept methadone [patients]." Societal and institutional stigma, reflected in the political commitment and resources available to harm reduction programs, client positioning within the health care system and attitudes of health care professionals can pose significant barriers to the accessibility of MMT and other harm reduction programs for opioid dependent individuals [4,66]. As Keane notes:

Prohibitionist policies threaten the freedom of users, damage their health and constitute them as marginal and stigmatized subjects excluded from normative categories of citizenship such as 'the general public' (p.229) [67]

Participant experiences of health care in this study were not influenced by one dimension of inequity, rather they were influenced by differential access to the social determinants of health and related multiple intersecting dimensions such as racism, classism, abilism and so on - dimensions that intersect with dominant ideologies regarding drug use and attendant assumptions, stereotypes and values. As Benoit notes, "[t]hose who face serious health concerns and at the same time are subject to multiple stigmas by virtue of their age, sex, gender, sexual orientation, race, ethnicity, socioeconomic or other social determinants, are less likely to access key resources and therefore differentially positioned to buffer themselves against the damaging impact of intersecting stigmas" (p. 5)[26].

### Constrained Lives: Harm Reduction, MMT and Individual Agency

Although MMT supports access to other interventions (e.g., anti-retroviral therapies) and there can be numerous positive outcomes, some participants found MMT highly restrictive; individual choice and freedom were limited by the policies and practices attached to MMT. As Young notes in her examination of the notion of 'inequality,' institutional structures and processes (including institutional rules and policies) "can inhibit the capacities of some people" at the same time as they expand the options of others (p.10) [68]. Many of the participants in our study described the ways in which their lives have been constrained by MMT. Individual agency was affected in several ways. Limits were placed on the freedom of some people to move from one area to another and choices were limited by power inequities. For example, several of the women in the study had children, who had been apprehended by the state as a consequence of the complex intersections of poverty, gender and problematic drug use and attendant social circumstances such as difficulties accessing safe housing; they described difficulty visiting their children because they could not access enough methadone (carries) to make the trip i.e., they were on daily doses of methadone *and/or *they could not access a pharmacy that dispensed methadone where their children were living, and/or they could not afford reliable transportation (sometimes needing to hitchhike) to see their children.

Although many people (Aboriginal and non-Aboriginal) experience the effects of the limits placed on agency through restrictive guidelines regarding MMT, Aboriginal experiences of MMT are impacted by sociopolitical factors that are unique to their experience. For example, Aboriginal children represent approximately 40% of the 76,000 children and youth placed in care in Canada [69] - a fact associated with poverty, problem substance use and inadequate housing [70](notably Aboriginal people only comprise 4-5% of the overall Canadian population). These conditions mediate the extent to which women report substance use patterns and access MMT and other harm reduction services. To provide effective and safe harm reduction, including MMT and other services, it is necessary to understand the social context(s) in which these experiences emerge [71,72].

In a similar but slightly different vein, several participants experienced MMT as being incompatible with a "normal" life and improved quality of life. In the following example, a client participant discusses such limitations,

... I'm going to be up there this summer or next summer [to see my relatives], but I'm on methadone right now so I have to get off the methadone, I'm only on twenty-two mls (milliliters) but by June I should be off.

A health care professional also discusses this issue in the following,

How can you travel with a drug habit? A raging drug habit... try and get that [methadone], it would be a nightmare to try and get that, some doctor in another province or something or other community to prescribe it, good luck... try and navigate that whole thing on your own...

For the client participant above and as the health professional notes, MMT can be highly constraining, including the lack of freedom to travel because of the inability of many to access methadone in other locales. However, what was also problematic in this case, as noted in a later discussion with this participant, was that MMT was not experienced as an informed choice. He believed he had been coerced by his doctor inappropriately; he perceived that he had used heroin minimally and now, six years later, he experienced MMT as seriously constraining - an experience shared by several other participants.

In keeping with the perspective of this client, a health care professional critiques the issue of "recruitment" to MMT as problematic in the following interview example, "I mean look at the methadone scene, I mean these drugs started to pop up all over not because they care for the people, [but because] there is money!" In our study, there was a general cynicism expressed regarding how MMT is being offered by some providers. Although most participants (clients and health care professionals) accepted MMT as a harm reduction approach, several believed that it was being used by some in power, such as a few "doctors... and pharmacists", as a means to make money "off of the backs of addicts." In our study, these views were fueled by a Canadian Broadcasting Corporation (CBC) news headline on September 11, 2008 that read, "Methadone kickbacks could lead to criminal investigation"; allegedly, several local pharmacies were reported to be paying "drug addicts" a fee each time they were dispensed methadone - money that was reportedly being used by some to buy illicit drugs [73]. In addition, the practice of charging daily dispensing fees rather than weekly dispensing fees ($15/day) was alleged to be the practice in some pharmacies, even though "weekly dispensing" was written on the prescription. The experience of 'being taken advantage of' because of being an "addict" in addition to the rules and regulations associated with MMT engendered a sense of vulnerability, and, to a belief by some participants, that they were being punished for their drug use. Although people with problematic substance use are not inherently vulnerable to stigma, they do face disadvantages relative to their ability to access resources and enact agency, i.e., enact control over their bodies and lives.

The "regime of control" has been reported elsewhere in the methadone literature in relation to random drug tests and urine screens that are used to ensure people using methadone are not "topping up" with illicit heroin or other drugs [74] as well as methadone consumption [25]; according to Vigilant, there is a 'felt' or 'perceived' stigma associated with these sorts of institutional regulations [74,27]- a perception that is created by policies that reinforce societal biases, e.g., those biases based in a moral stance against drug use, rather than those that focus on the sociopolitical and cultural context in which drug use occurs. For people most marginalized by social and structural inequity such as Aboriginal people, 'constrained lives' may make them the target of profound stigmatization that may appear as insurmountable because of other intersecting issues, poverty, homelessness and so on. In addition to the constraints posed by treatment itself, many of the participants in this study (79%) were also constrained by unstable housing and limited options related to same.

### Harm Reduction, MMT and Homelessness

Women and men whose poverty leads them to live in unsafe housing units in sections of the city where problematic drug use surrounds them, whose need for access to MMT and antiretroviral treatment leads to confinement to particular urban settings, and whose Aboriginality may further limit their housing choices within particular areas, exemplify the need to examine harm reduction and MMT using an intersectional analysis. An Aboriginal participant who was accessing MMT in our study describes his living arrangements in the following, "... Native housing, you know what, it's a real crack house right?... I wish I worked there, you know, at nights, I wish they hired me at nights not to let people in, I wouldn't." For this participant and many others, housing conditions acted as a barrier to positive outcomes. Here, an intersectional lens draws attention to the disturbing ways that homelessness, poverty, substance use and racialization intersect to exacerbate peoples' experiences of social suffering, i.e., to those human conditions with roots and consequences associated with social, economic and political power - suffering that is both created by the way power is inflicted on human experience and how this power shapes the response to it. As noted by Kleinman et al., "the trauma, pain and disorders to which atrocity gives rise [ongoing colonial processes and practices] are health conditions; yet they are also political and cultural matters" (p.ix) [63]. Another participant, an Aboriginal woman who lives with HIV illness, Hepatitis C and mental illness, describes her experience in the following,

There must be something wrong with me, I won't go shower, I take sponge baths in my room... the hotel is so skungy...we share a bathroom...like if its catchable...

For this woman, the hotel she was living in generated tremendous fear of further health compromise. The vermin and filth of the hotels where many of the participants in this study reside is well documented in other places [75]. Although the lives of the Aboriginal men and women with mental health illness on MMT who are living in poverty resemble those of other impoverished people, the intersection of poverty, mental illness, HIV/AIDS, Hepatitis C, and gender (as examples) brings with it a special set of circumstances and challenges to successful harm reduction. We argue that intersections across these multiple axes of differentiation do not have additive effects; rather the findings of our study suggest that peoples' experiences, although similar across some dimensions, are differentiated by the disadvantages (and advantages) posed by their location across these axes.

## Conclusions

Harm reduction, including MMT, "driven solely by reducing the harm of drug use is not sufficient to address inequities in health and access to health care for those who are street involved" (p.8) [4]. As Pauly notes, the root causes of problematic substance use must be addressed in conjunction with the social determinants of health [4], determinants such as stigma. The harms that emanate from drug policy and health policy must also be considered.

Regardless of the intent of health care providers, stigma and discrimination were experienced by the participants in our study in everyday attempts to access mental health and addictions services, including harm reduction services. In keeping with the perspective posed by Stuber et al. [64], our research points to the need for more work to be done to fully understand the often unintentional impact of stigma and discrimination as social processes linked to the reproduction of inequality and exclusion, and the many ways in which stigma and discrimination affect persons marginalized by social and structural inequity, including the possible negative consequences related to health and well-being. As Rossiter and Morrow argue, "the adoption of an intersectional perspective and anti-oppression framework in anti-stigma and discrimination work will both allow for greater understanding and awareness of intersecting social identities and the layering of stigma and discrimination, and promise better outcomes for the reduction of stigma and discrimination at both social and structural levels" [14]. In addition, as Lloyd notes, the entrenched and widely held view that persons who use drugs are solely culpable for their condition needs to be addressed [27]; people, including health professionals and the media regarding the causes and nature of addiction.

People's lives were also constrained by the way in which services were offered. Employment opportunities, access to children, attachments to family and community in other geographic locations and so on, were constrained by treatment. In our research, we have found that some women's capacity to parent is limited by MMT policies regarding carries and social housing policies related to children. To determine the constellation of risks for a woman in the context of being, as one example, a single mother in MMT, Aboriginal, unemployed and homeless or near homeless, we need to explore how and where these identities intersect to shape this woman's personal experience. As Collins et al. discuss, in the context of research examining the constellation of intersecting risks for inner city women with severe mental illness [8], we must understand the multiple systems of power at work in women's lives.

Lastly, in this study, most of the participants were living in unstable housing or were homeless. We define homelessness in much the same way as Patterson et al. [76] to include both the absolutely ("street") homeless as well as those at imminent risk of homelessness. The paths in and out of homelessness usually involve some form of inadequate housing. In addition, "while the most visible homeless individuals are those living on the streets, many more individuals are precariously housed in rooming houses, transitional housing, substandard rental suites, shacks and cabins without running water, and other forms of substandard or unaffordable housing" - those individuals who are both inadequately housed and inadequately supported are particularly at-risk for homelessness (p. 17) [76]. Absolute homelessness refers to those without any physical shelter. Housing is considered an important social determinant of health and housing for Aboriginal peoples is notably lagging in comparison to non-Aboriginal people in both urban and rural settings. For example, it is estimated that 41% of all Aboriginal peoples in British Columbia (BC), Canada are at-risk of homelessness and 23% are absolutely homeless [76,77]. People with severe addictions and/or mental illness also can be found in this group - they make up anywhere from 33% to over 60% of the overall homeless population [76].

Although harm reduction is not a panacea and it is not feasible to believe that it will address all social oppressions, as Boyd notes, "harm reduction initiatives can provide a shift in policy and practice that bring social factors to the foreground. It can also pave the way for compassionate health and human-rights models of care, and the rejection of drug policy based on punitive ideology" (p.5) [78]. However, harm reduction must move beyond a narrow concern with the harms directly related to drugs and drug use practices to address the harms associated with the determinants of drug use, such as homelessness, and the harms of drug and health policy.

To consider long-term structural change in broad social systems is a daunting task, but operating from a social justice framework, it is one that we see as essential to making any substantial headway to address health disparities. Concerted political action as well as the forging of alliances across the domains of many groups - policy makers; researchers working from multiple paradigms which include participatory and community-based approaches; the media; grassroots activists; professional organizations; and most importantly, community groups, are needed to bring about the kinds of change necessary to reduce health disparities [6,12].

Pauly argues for harm reduction approaches/interventions that integrate more fully with "primary health care and the social determinants of health within a social justice framework" (p.8) [4]. In addition, we argue for relational practices that mitigate the effects of social inequity and address mental health and addictions services, including harm reduction - practices that reflect an understanding of the ways in which health and well-being (and health care) are shaped by the contextual features of peoples' lives [79]. Harm reduction tools, including MMT, need to reflect an understanding that systems of power/oppression that operate across the axes of race, class, gender, ability and so on, are interlocking; to focus on drug use to the exclusion of other factors is problematic.

## List of Abbreviations

MMT: Methadone Maintenance Treatment; PTSD: Post Traumatic Stress Disorder; RNAO: Registered Nurses Association of Ontario.

## Conflict of interests

The authors declare that they have no competing interests.

## Authors' contributions

VS was the principle investigator on the study, designed and participated in all aspects of the study, including the data analysis and interpretation of the data and drafted the manuscript. AJB was a co-investigator, assisted in the design and in all aspects of the study, including the data analysis and interpretation of the data and assisted with the drafting of the manuscript. CV assisted in the interpretation of the data and the drafting of the manuscript. VJ participated in data analysis and interpretation of the data and assisted with the final draft of the manuscript. All authors read and approved the final manuscript.

## References

[B1] International Federation of Red Cross and Red Crescent Societies (IFRC) Spreading the light of science: Guidelines on harm reduction related to injecting drug use 2003 Geneva: International Federation of Red Cross and Red Crescent Societies

[B2] Wood E Kerr T Small W Li K Marsh D Montaner JS Tyndall MW Changes in public order after the opening of a medically supervised safer injecting facility for illicit injection drug users Can Med Assoc J 2004 171 731 734 10.1503/cmaj.1040774 PMC517857 15451834

[B3] Wood E Tyndall MW Montaner JS Kerr T Summary of findings from the evaluation of a pilot medically supervised safer injecting facility Can Med Assoc J 2006 175 1399 1404 10.1503/cmaj.060863 PMC1635777 17116909

[B4] Pauly B Harm reduction through a social justice lens Int J Drug Policy 2008 19 4 10 10.1016/j.drugpo.2007.11.005 18226520

[B5] Pauly B Goldstone I McCall J Gold F Payne S The ethical, legal and social context of harm reduction Can Nurse 2007 103 19 23 17990401

[B6] Hankivsky O Cormier R DeMerich D Intersectionality: Moving women's health research and policy forward 2009 Vancouver: Women's Health Research Network21114082

[B7] Bannerji H Thinking Through: Essays on Feminism, Marxism, and Anti-Racism 1995 Toronto, ON, Canada: Women's Press

[B8] Collins PY von Unger H Ambrister A Church ladies, good girls, and locas: Stigma and the intersection of gender, ethnicity, mental illness, and sexuality in relation to HIV risk Soc Sci Med 2008 67 389 397 10.1016/j.socscimed.2008.03.013 18423828 PMC2587415

[B9] Crenshaw KW Fineman MA, Mykitiuk R Mapping the margins: Intersectionality, identity politics, and violence against women of color The Public Nature of Private Violence 1994 New York: Routledge 93 118

[B10] McCall L The complexity of intersectionality Signs 2005 30 1771 1800 10.1086/426800

[B11] Anderson JM Reimer Kirkham S Coward H, Ratanakul P Discourses on health: A critical perspective A Cross-Cultural Dialogue on Health Care Ethics 1999 Waterloo, ON, Canada: Wilfred Laurier University Press 47 67

[B12] Weber L Parra-Medina D Intersectionality and women's health: Charting a path to eliminating disparities Advances in Gender Research 2003 7 181 230

[B13] Cole E Intersectionality and research in psychology American Psychologist 2009 64 10 180 10.1037/a0014564 19348518

[B14] Rossiter K Morrow M Hankivsky O Intersectional frameworks in mental health: Moving from theory to practice Health Inequities in Canada: Intersectional Frameworks and Practices 2011 Toronto: UBC Press 312 330

[B15] Reist D Marlatt GA Goldner E Parks GA Fox J Kang S Dive L Every door is the right door: A British Columbia planning framework to address problem substance use and addiction 2004 Victoria, BC: Author

[B16] College of Physicians and Surgeons of Ontario (CPSO) Methadone Maintenance Guidelines 2005 http://www.cpso.on.ca/uploadedFiles/policies/guidelines/methadone/Meth%20Guidelines%20_Oct07.pdf

[B17] Registered Nurses Association of Ontario (RNAO) Supporting Clients on Methadone Maintenance Treatment: Clinical Best Practice Guidelines 2009 Toronto: Registered Nurses' Association of Ontario

[B18] Health Canada Best Practices Methadone Maintenance Treatment 2002 http://www.hc-sc.gc.ca/hc-ps/alt_formats/hecs-sesc/pdf/pubs/adp-apd/methadone-bp-mp/methadone-bp-mp-eng.pdf

[B19] Centre for Addiction and Mental Health (CAMH) Mental health and addictions 101 series 2008 http://www.camh.net/education/Online_courses_webinars/mha101/index.html

[B20] Krüsi A Wood E Montaner J Kerr T Social and structural determinants of HAART access and adherence among injection drug users International Journal of Drug Policy 2010 21 4 9 10.1016/j.drugpo.2009.08.003 19747811

[B21] Reist D Methadone maintenance treatment in British Columbia, 1996-2008: Analysis and recommendations 2010 Victoria, BC: Ministry of Healthy Living and Sport and the Centre for Addictions Research in BC, University of Victoria

[B22] Goffman E Stigma: Notes on the Management of Spoiled Identity 1963 Englewood Cliffs, New Jersey: Prentice-Hall

[B23] Link BG Phelan JC Conceptualizing stigma Annual Review of Sociology 2001 27 363 85 10.1146/annurev.soc.27.1.363

[B24] Link BG Phelan J Stigma and its public health implications Lancet 2006 367 528 29 10.1016/S0140-6736(06)68184-1 16473129

[B25] Anstice S Strike CJ Brands B Supervised methadone consumption: Client issues and stigma Substance Use and Misuse 2009 44 794 808 10.1080/10826080802483936 19444722

[B26] Benoit C Shumka L Barlee D Stigma and the health of vulnerable women Women's Health Research Network 201021726038

[B27] Lloyd C Sinning and sinned against: The stigmatisation of problem drug users 2010 London: The UK Drug Policy Commission (UKDPC)

[B28] Keusch GT Wilentz J Kleinman A Stigma and global health: Developing a research agenda Lancet 2006 367 525 27 10.1016/S0140-6736(06)68183-X 16473128

[B29] Gardner CB Gronfein WP Reflections on varieties of shame induction, shame management, and shame avoidance in some works of Erving Goffman Symbolic Interaction 2005 28 175 182 10.1525/si.2005.28.2.175

[B30] Poole N Gender does matter: Coalescing on women and substance use Cross Currents 2007 http://www.camh.net/Publications/Cross_Currents/Spring%202007/genderdoesmatter_spring07crcu.html

[B31] Salmon A Poole N Morrow M Greaves L Ingram R Pederson A Improving conditions: Integrating sex and gender into federal mental health and addictions policy 2006 Vancouver: The British Columbia Centre of Excellence for Women's Health

[B32] Cheung YW Substance abuse and developments in harm reduction Can Med Assoc J 2000 162 1697 1700 PMC1232508 10870502

[B33] Marlatt GA Blume AW Parks GA Integrating harm reduction therapy and traditional substance abuse treatment J Psychoactive Drugs 2001 33 13 21 11332996 10.1080/02791072.2001.10400463

[B34] Thomas G Assessing the need for prison-based needle exchange programs in Canada: A situational analysis 2005 Ottawa: Canadian Centre on Substance Abuse

[B35] Amato L Davoli M Perucci C Ferri M Faggiano F Mattick R An overview of systematic reviews of the effectiveness of opiate maintenance therapies: Available evidence to inform clinical practice and research Journal of Substance Abuse Treatment 2005 28 321 329 10.1016/j.jsat.2005.02.007 15925266

[B36] Mattick R Breen C Kimber J Davoli M Methadone maintenance therapy versus no opioid replacement therapy for opioid dependence Cochrane Database of Systematic Reviews 2009 1 CD002209 10.1002/14651858.CD002209 12519570

[B37] Mattick R Kimber J Breen C Davoli M Buprenorphine maintenance versus placebo or methadone maintenance for opioid dependence Cochrane Database of Systematic Reviews 2009 1 CD002207 10.1002/14651858.CD002207.pub2 15266465

[B38] Caplehorn J Dalton M Cluff M Petrenas AM Retention in methadone maintenance and heroin addicts' risk of death Addiction 1994 89 203 207 10.1111/j.1360-0443.1994.tb00879.x 8173486

[B39] Canadian AIDS Society Harm reduction and substance use: A position statement 2000 http://www.cdnaids.ca/harmreductionandsubstanceuseadopted

[B40] Adelson N The embodiment of inequity: Health disparities in Aboriginal Canada Can J Public Health 2005 96 Suppl 2 S45 S61 16078555 10.1007/BF03403702 PMC6975716

[B41] Amnesty International Stolen sisters: A human rights response to discrimination and violence against indigenous women in Canada 2004 http://www.amnesty.ca/campaigns/sisters_overview.php

[B42] Brzozowski JA Taylor-Butts A Johnson S Victimization and offending among the Aboriginal population in Canada Juristat 2006 26 1 31

[B43] Jacobs K Gill K Substance abuse in an urban Aboriginal population: Social, legal, and psychological consequences J Ethn Subst Abuse 2002 1 7 25

[B44] Royal Commission on Aboriginal Peoples (RCAP) Guidelines for ethical research with Aboriginal peoples 1993 Ottawa: RCAP

[B45] Schnarch B Ownership, control, access, and possession (OCAP) or self-determination applied to research: A critical analysis of contemporary First Nations research and some options for First Nations communities 2004 First Nations Centre, National Aboriginal Health Organization

[B46] Anderson JM Perry J Blue C Browne AJ Henderson A Lynam J Kirkham Reimer S Semeniuk P Smye V "Re-writing" cultural safety within the postcolonial and postnationalist feminist project: Toward new epistemologies of healing Advs in Nurs Sci 2003 26 196 214 10.1097/00012272-200307000-00005 12945655

[B47] Sandelowski M Qualitative analysis: What it is and how to begin? Research in Nursing & Health 1995 18 371 375 10.1002/nur.4770180411 7624531

[B48] Chrisjohn R Young S Maraun M The Circle Games: Shadows and Substance in the Indian Residential School Experience in Canada 1997 Penticton: Theytus Books Ltd

[B49] Kelm M Colonizing Bodies: Aboriginal Health and Healing in British Columbia 1900-50 1998 Vancouver: University of British Columbia Press

[B50] Royal Commission on Aboriginal Peoples Report of the Royal Commission on Aboriginal peoples: Volume 3, gathering strength 1996 Ottawa: Royal Commission on Aboriginal Peoples

[B51] Waldram JB The Way of the Pipe: Aboriginal Spirituality and Symbolic Healing in Canadian Prisons 1997 Peterborough: Broadview Press

[B52] Waldram JB Herring DA Young TK Aboriginal Health in Canada: Historical, Cultural, and Epidemiological Perspectives 2006 2 Toronto: University of Toronto Press

[B53] Warry W Ending Denial: Understanding Aboriginal Issues 2009 Toronto: University of Toronto Press

[B54] Indian Residential Schools Settlement http://aabc.ca/newsletter/17_2/aabc_newsletter_indian_residential_schools.htm

[B55] Reimer Kirkham S The politics of belonging and intercultural health care provision West J Nurs Res 2003 25 762 780 10.1177/0193945903256709 14596178

[B56] Parkes T British Columbia methadone maintenance treatment program: A qualitative systems review (1996-2008) 2009 Victoria: University of Victoria

[B57] Luoma JB Twohig MP Waltz T Hayes SC Roget N Padilla M Fisher G An investigation of stigma in individuals receiving treatment for substance abuse Addictive Behaviors 2007 32 1331 1346 10.1016/j.addbeh.2006.09.008 17092656

[B58] Butters J Erickson PG Meeting the health care needs of female crack users: A Canadian example Women Health 2003 37 1 17 12839304 10.1300/J013v37n03_01

[B59] Pauly B Shifting moral values to enhance access to health care: Harm reduction as a context for ethical nursing practice Int J Drug Policy 2008 19 195 204 10.1016/j.drugpo.2008.02.009 18467086

[B60] Furniss E The Burden of History: Colonialism and the Frontier Myth in a Rural Canadian Community 1999 Vancouver: University of British Columbia Press

[B61] Butt G Paterson B McGuinness L Living with the stigma of hepatitis C Western Journal of Nursing Research 2008 30 204 221 17630381 10.1177/0193945907302771

[B62] Treloar C Rhodes T The lived experience of Hepatitis C and its treatment among injection drug users: Qualitative synthesis Qual Health Res 2009 19 1321 1334 10.1177/1049732309341656 19690211

[B63] Kleinman A Das V Lock M Kleinman A, Das V, Lock M Introduction Social Suffering 1997 Berkeley: University of California Press ix xxvii

[B64] Stuber J Meyer I Link B Stigma, prejudice, discrimination and health Soc Sci Med 2008 67 351 357 10.1016/j.socscimed.2008.03.023 18440687 PMC4006697

[B65] Padilla M Castellanos D Guilamo-Ramos V Reyes AM Sànchez Marte LE Soriano MA Stigma, social inequality, and HIV risk disclosure among Dominican male sex workers Soc Sci Med 2008 67 380 388 10.1016/j.socscimed.2008.03.014 18410986 PMC2928563

[B66] Public Health Agency of Canada Harm reduction and injection drug use: An international comparative study of contextual factors influencing the development and implementation of relevant policies and programs 2003 http://www.phac-aspc.gc.ca/hepc/pubs/hridu-rmudi/index-eng.php

[B67] Keane H Critiques of harm reduction, morality and the promise of human rights Int J Drug Policy 2003 14 227 232 10.1016/S0955-3959(02)00151-2

[B68] Young IM Equality of Whom? Social groups and judgments of injustice J Polit Philos 2001 9 1 18 10.1111/1467-9760.00115

[B69] Farris-Manning C Zandstra M Children in Care in Canada: Summary of current issues and trends and recommendations for future research 2003 Child Welfare League of Canada, Ottawa http://www.cecw-cepb.ca/publications/574

[B70] Blackstock C Trocmé N Ungar M Community based child welfare for aboriginal children: Supporting resilience through structural change [2004] Pathways to Resilience: A Handbook of Theory, Methods and Interventions Thousand Oaks, CA: Sage in press

[B71] Poole N Urquhart C Pregnant women and alcohol: We need to move from stigma to support Visions Journal 2006 2 16 17

[B72] Salmon A Dis/abling states, Dis/abling citizenship: Young Aboriginal mothers, substantive citizenship, and the medicalization of FAS/FAE Journal of Critical Education Policy 2007 5

[B73] CBC News: Methadone kickbacks could lead to criminal investigation http://www.cbc.ca/news/canada/british-columbia/story/2008/09/11/bc-methadone-kickbacks-investigation-downtown-eastside.html

[B74] Vigilant LG The Stigma Paradox in Methadone Maintenance: Naive and Positive Consequences of a 'Treatment Punishment' Approach to Opiate Addiction Humanity and Society 2004 28 403 418

[B75] Maté G In the Realm of Hungry Ghosts: Close Encounters with Addiction 2009 Toronto: Vintage Canada

[B76] Patterson M Somers JM McIntosh K Shiell A Frankish CJ Housing and support for adults with severe addiction and/or mental illness in British Columbia 2007 http://www.carmha.ca/publications/documents/Housing-SAMI-BC-FINAL-PD.pdf

[B77] United Native Nations Society Aboriginal homelessness in British Columbia 2001 http://www.urbancenter.utoronto.ca/pdfs/elibrary/UNNS_Aboriginal_Homelessn.pdf

[B78] Boyd SC The Journey to Compassionate Care: One Woman's Experience with Early Harm-Reduction Programs in BC 2007 Canadian Women's Health Network http://www.cwhn.ca/en/node/39390 Retrieved February 19, 2010

[B79] Doane GH Varcoe C Family Nursing as Relational Inquiry: Developing a Health-promoting Practice 2005 Philadelphia Lippincott, Williams & Wilkins

